# Supplementary material for: Simultaneous triple-parametric optical mapping of transmembrane potential, intracellular calcium and NADH for cardiac physiology assessment
Source: Commun Biol. 2022 Apr 6;5:319. doi: 10.1038/s42003-022-03279-y (PMC8987030; doi:10.1038/s42003-022-03279-y)
Supplement: Supplementary file 2 — Description of Additional Supplementary Files [file 42003_2022_3279_MOESM2_ESM.pdf]

Description of Additional Supplementary Files

File name: Supplementary Data 1
Description: Modulation of cardiac physiology by Blebbistatin (15 mM), 4-AP (7 mM) and Verapamil (1 mM).

File name: Supplementary Data 2
Description: Modulation of cardiac physiology by Ischemia and Reperfusion.
