# Supplementary material for: Simultaneous triple-parametric optical mapping of transmembrane potential, intracellular calcium and NADH for cardiac physiology assessment
Source: Commun Biol. 2022 Apr 6;5:319. doi: 10.1038/s42003-022-03279-y (PMC8987030; doi:10.1038/s42003-022-03279-y)
Supplement: Supplementary file 5 — Reporting Summary [file 42003_2022_3279_MOESM5_ESM.pdf]

nature portfolio

Corresponding author(s): Sharon A George, Igor R Efimov

Last updated by author(s): 02/21/2022

Reporting Summary

Nature Portfolio wishes to improve the reproducibility of the work that we publish. This form provides structure for consistency and transparency in reporting. For further information on Nature Portfolio policies, see our Editorial Policies and the Editorial Policy Checklist.

Statistics

For all statistical analyses, confirm that the following items are present in the figure legend, table legend, main text, or Methods section.

n/a	Confirmed	
☐	☒	The exact sample size (n) for each experimental group/condition, given as a discrete number and unit of measurement
☐	☒	A statement on whether measurements were taken from distinct samples or whether the same sample was measured repeatedly
☐	☒	The statistical test(s) used AND whether they are one- or two-sided<br>*Only common tests should be described solely by name; describe more complex techniques in the Methods section.*
☒	☐	A description of all covariates tested
☐	☒	A description of any assumptions or corrections, such as tests of normality and adjustment for multiple comparisons
☐	☒	A full description of the statistical parameters including central tendency (e.g. means) or other basic estimates (e.g. regression coefficient) AND variation (e.g. standard deviation) or associated estimates of uncertainty (e.g. confidence intervals)
☐	☒	For null hypothesis testing, the test statistic (e.g. F, t, r) with confidence intervals, effect sizes, degrees of freedom and P value noted<br>*Give P values as exact values whenever suitable.*
☒	☐	For Bayesian analysis, information on the choice of priors and Markov chain Monte Carlo settings
☒	☐	For hierarchical and complex designs, identification of the appropriate level for tests and full reporting of outcomes
☒	☐	Estimates of effect sizes (e.g. Cohen's d, Pearson's r), indicating how they were calculated

Our web collection on statistics for biologists contains articles on many of the points above.

Software and code

Policy information about availability of computer code

Data collection	BV Workbench software from SciMedia was used for data acquisition. It is available at the vendors website.
Data analysis	Rhythm 3.0 which is an updated version of Rhythm 1.2, an open-source software published by our group, was used for data analysis. It is published on Github (https://github.com/optocardiography).

For manuscripts utilizing custom algorithms or software that are central to the research but not yet described in published literature, software must be made available to editors and reviewers. We strongly encourage code deposition in a community repository (e.g. GitHub). See the Nature Portfolio guidelines for submitting code & software for further information.

Data

Policy information about availability of data

All manuscripts must include a data availability statement. This statement should provide the following information, where applicable:

- Accession codes, unique identifiers, or web links for publicly available datasets
- A description of any restrictions on data availability
- For clinical datasets or third party data, please ensure that the statement adheres to our policy

The data analysis software is available as an open source software on Github. All data will also be available upon request to the corresponding authors.

Field-specific reporting

Please select the one below that is the best fit for your research. If you are not sure, read the appropriate sections before making your selection.

☒ Life sciences ☐ Behavioural & social sciences ☐ Ecological, evolutionary & environmental sciences

For a reference copy of the document with all sections, see nature.com/documents/nr-reporting-summary-flat.pdf

Life sciences study design

All studies must disclose on these points even when the disclosure is negative.

Sample size	Sample size was determined by performing Power analysis using estimates of the difference between means (M) and the standard deviation (SD) of the endpoint variables (CV and APD). These preliminary data regarding estimated SD and M has been previously collected by our laboratory. The statistical power was fixed to 80% in order to maximize the reliability of the statistical results. The following equation was used for the calculation of sample size: n = 2*[SD*Z/M]^2, where Z is determined by the Gaussian distribution of the endpoint variable (=2.32 for an ideal statistical power reaching 80%. We used a sample size of 5 hearts for the drug testing studies and another 5 hearts for the ischemia studies which is sufficient for these studies.
Data exclusions	No data were excluded from this study.
Replication	The studies (drug testing and ischemia) was performed in 5 separate mouse hearts each (total of 10 hearts). The technique and observed results were reproducible in all 10 hearts.
Randomization	Mice were randomly allocated to each application tested in this study (drug testing and ischemia). Male and female mice were used at roughly equal proportions.
Blinding	Not relevant. Mice on the same strain/background were used in all experiments in this study. No sex specific differences in acute response to these drugs/conditions are expected.

Reporting for specific materials, systems and methods

We require information from authors about some types of materials, experimental systems and methods used in many studies. Here, indicate whether each material, system or method listed is relevant to your study. If you are not sure if a list item applies to your research, read the appropriate section before selecting a response.

Materials & experimental systems

n/a	Involved in the study	
☒	☐	Antibodies
☒	☐	Eukaryotic cell lines
☒	☐	Palaeontology and archaeology
☐	☒	Animals and other organisms
☒	☐	Human research participants
☒	☐	Clinical data
☒	☐	Dual use research of concern

Methods

n/a	Involved in the study	
☒	☐	ChIP-seq
☒	☐	Flow cytometry
☒	☐	MRI-based neuroimaging

Animals and other organisms

Policy information about studies involving animals; ARRIVE guidelines recommended for reporting animal research

Laboratory animals	C56BL/6 mice of both sexes were used at 20-30 weeks of age.
Wild animals	The study did not involve wild animals.
Field-collected samples	The study did not involve field collected samples.
Ethics oversight	All experimental protocols were approved by the Institutional Animal Care and Use Committee at the George Washington University and are in accordance with the NIH Guide for the Care and Use of Laboratory Animals.

Note that full information on the approval of the study protocol must also be provided in the manuscript.
